# Case Report: Pericardial tamponade during left ventricular radiofrequency ablation with spontaneous hemostasis

**DOI:** 10.3389/fcvm.2025.1669648

**Published:** 2025-10-29

**Authors:** Changjian He, Jinming Lin, Chunhua Ding, Wenchang Zhang

**Affiliations:** Cardiac Department, Aerospace Center Hospital, Peking University Aerospace School of Clinical Medicine, Beijing, China

**Keywords:** pericardial tamponade, left ventricular premature, radiofrequency ablation, spontaneous hemostasis, pericardial puncture

## Abstract

**Background:**

Pericardial tamponade is a rare but life-threatening complication of catheter ablation for ventricular arrhythmias. While overt perforation—often steam-pop related—is classically implicated, alternative bleeding mechanisms are less well defined.

**Case:**

A 66-year-old man underwent left ventricular (LV) ablation for frequent idiopathic premature ventricular beats (34.3%). RF energy (30–40 W, ≤90 s) at the LV anterior wall markedly suppressed ectopy, followed by abrupt chest discomfort and hypotension. Fluoroscopy suggested pericardial effusion; emergent pericardiocentesis yielded 150 ml of bright red, arterialized blood with rapid hemodynamic recovery. Biventricular angiography showed no contrast extravasation, and serial echocardiography confirmed no recurrent effusion. The patient stabilized with indwelling drainage and was discharged uneventfully.

**Conclusion:**

Tamponade during LV ablation may occur without overt perforation. Prompt recognition and targeted drainage can be definitive when bleeding is self-limited.

## Background

Pericardial tamponade is one of the complications of ventricular radiofrequency ablation. In pericardial tamponade, blood compresses the cardiac chambers, leading to hemodynamic impairment, circulatory shock, cardiac arrest, and death (1). Early detection and diagnosis of pericardial tamponade are critical for effective management of these patients. Cardiac perforation leading to tamponade is a common cause of pericardial tamponade during radiofrequency ablation, with an incidence of approximately 1%–2% (2). Most perforations are caused by steam pop phenomenon, which refers to barotrauma resulting from gas production due to excessive heating during catheter ablation or complications associated with transseptal perforation. However, radiofrequency ablation-related pericardial tamponade can also occur without explicit cardiac perforation, and may be characterized by bleeding resulting from minor injuries to the ventricular wall and/or epicardial small vessels. Here, we present a rare case of pericardial tamponade during radiofrequency ablation for idiopathic left ventricular premature beats without definite cardiac perforation and with spontaneous hemostasis.

## Case report

**Figure 1:**
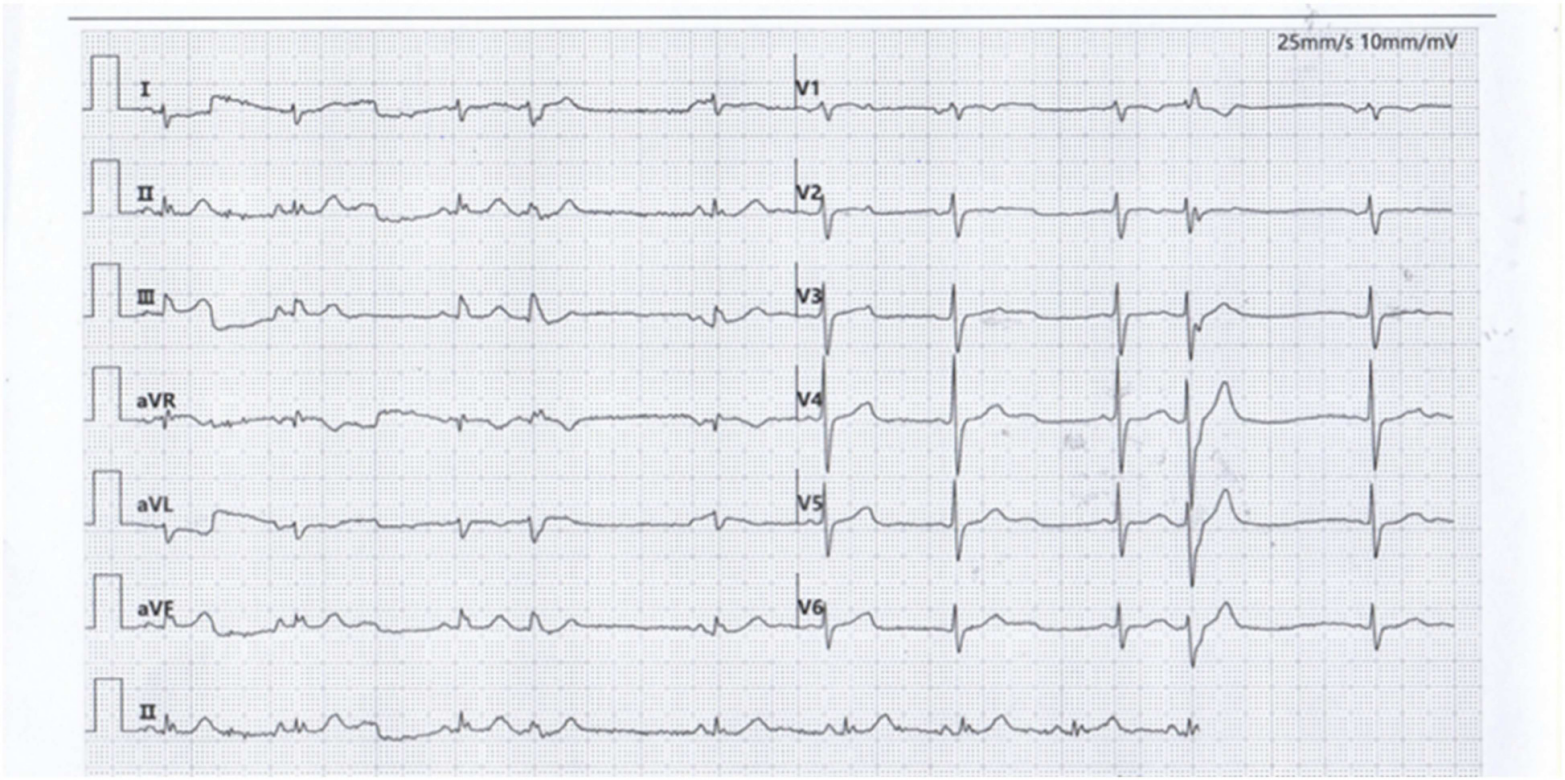
Premature ventricular contraction surface electrocardiogram.

The patient was a 66-year-old male referred to our medical institution due to palpitations. He was diagnosed with idiopathic ventricular premature beats (VPBs) via ambulatory electrocardiogram (ECG) recording, with a VPB burden of 34.3% (26,949/78,629). The patient had no history of organic heart disease, diabetes mellitus, chronic kidney disease, or coronary artery disease. Medical therapy for VPB control was limited, so catheter radiofrequency ablation for VPBs was performed. Preoperative ECG morphology of VPBs suggested the origin of premature beats was the left ventricle (LV) (Figure 1). The VPBs showed a right bundle branch block (RBBB) morphology with transition starting at V2, suggesting a possible origin from the LV apex. The preoperative cardiac ultrasound and left ventricular myocardial contrast echocardiography did not indicate any abnormalities in the left heart structure (Supplementary Figures SA,B).

After obtaining written informed consent from the patient, electrophysiological examination was performed, with continuous invasive arterial blood pressure monitoring via an arterial access. Under local anesthesia, the left femoral vein was punctured for his bundle electrode placement. Subsequently, the right femoral artery was punctured. Then intravenous heparin 5,000 IU was administered at the beginning of the procedure. A TCSE saline-irrigated tip catheter was advanced retrogradely through the aorta into the left ventricle for mapping and ablation using the Ensite three-dimensional mapping system. The earliest activation site of ventricular premature beats was identified at the apical portion of the left ventricular anterior wall (see target map), where radiofrequency (RF) energy was applied to terminate the premature beats.

### Target map

**Figure 2:**
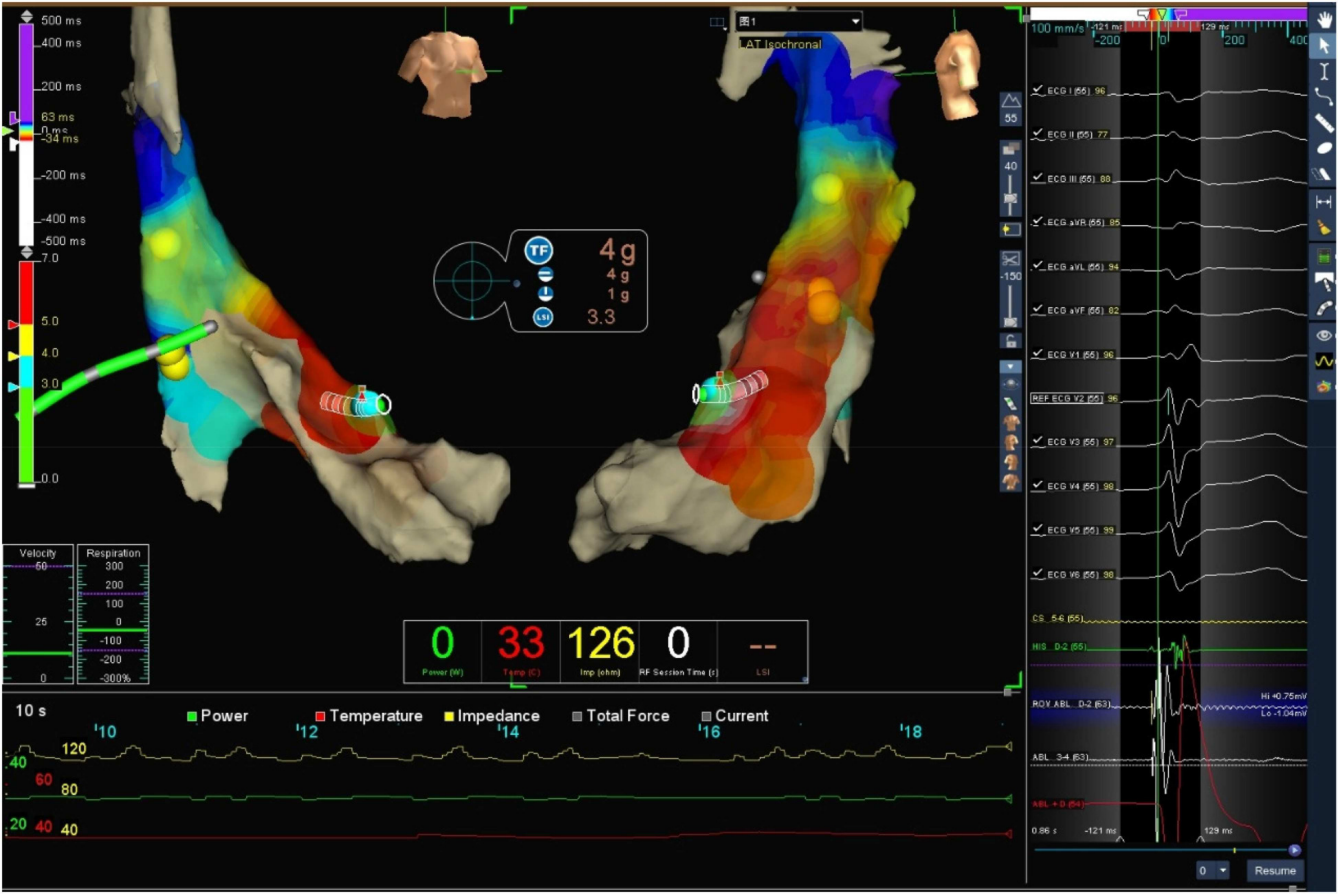
Target three-dimensional mapping image and ablation parameter curve graph: the green curve represents power, red represents temperature, and yellow represents impedance.

**Figure 3:**
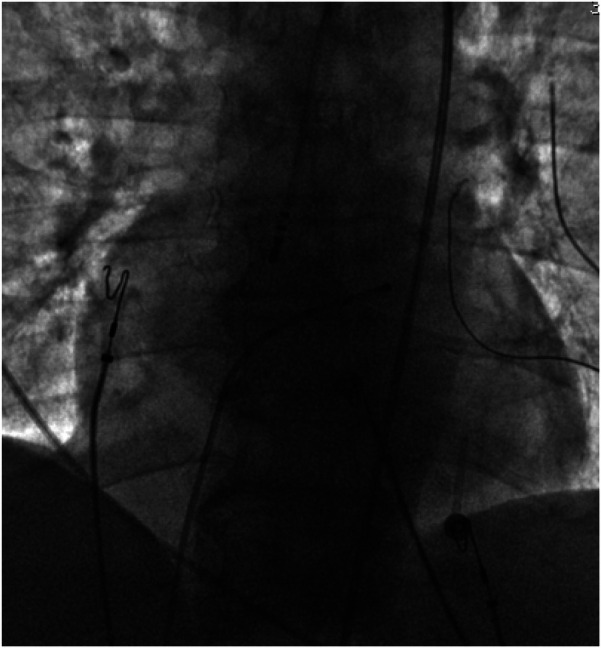
Pericardial effusion.

**Figure 4:**
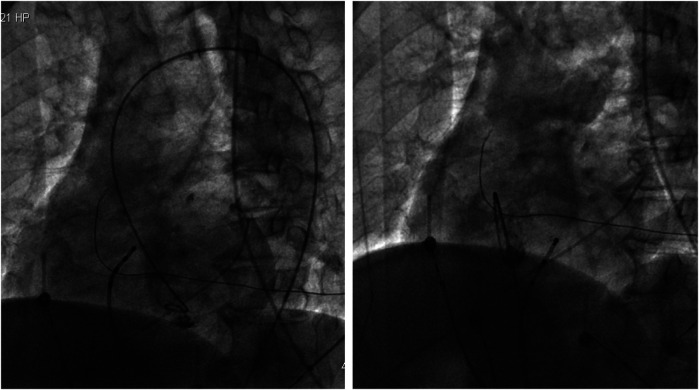
**(Left)** Left ventricular angiography showing no contrast agent extravasation into the pericardial cavity. **(Right)** Right ventricular angiography showing no contrast agent extravasation into the pericardium.

With the radiofrequency ablation power set at 30–40 W and the target temperature at 55 °C, the radiofrequency transmission lasted up to 90 s (Figure 2). During the ablation process, steam pop was not perceived, and there were no sudden changes in ablation parameters such as impedance、power or pressure values (Figure 2). After the termination of radiofrequency, the ventricular premature beats were significantly reduced. During the observation period after ablation (not immediately after), the patient suddenly complained of chest tightness, shortness of breath, and chest pain, and the blood pressure dropped to 102/65 mmHg (baseline blood pressure value: 149/78 mmHg). x-ray fluoroscopy observation showed a translucent shadow around the heart in the pericardial cavity (Figure 3), and the patient was suspected of having pericardial tamponade. Pericardial puncture and drainage were immediately performed. After slowly withdrawing 150 ml of bright red blood, no more blood was withdrawn, and the patient's blood pressure gradually recovered and tended to be stable. The ACT value immediately before pericardiocentesis was 126 s. Protamine was not administered and no additional heparin was administered due to the short duration of ablation. The unexpectedly low ACT at the time of pericardiocentesis may reflect partial heparin metabolism and consumption. Blood gas analysis of the red blood cells drawn from the pericardium suggested arterial blood. Under fluoroscopy, the translucent zone around the heart was smaller than before. In order to further clarify the perforation site, angiography was performed in the left and right ventricles(Supplementary Videos S1–S4), and it was found that the contrast agent did not extravasate outside the cardiac cavity (Figures 4A,B). Bedside transthoracic echocardiography showed that the pericardial effusion did not further increase. After indwelling the pericardial drainage tube, the patient was returned to the cardiac care unit for further observation. After 1 day of continuous drainage, no effusion was drained, and continuous review of echocardiography showed no obvious effusion (Supplementary Figures C,D). The patient was discharged after the drainage tube was removed.

## Discussion

Radiofrequency ablation (RFA) has long been an integral part of clinical practice and has become a critical therapeutic option for most clinically relevant arrhythmias. Achieving an optimal balance between the efficacy and safety of RFA is widely recognized as a significant challenge (3). Despite advances in the understanding of arrhythmias, accumulated experience with radiofrequency ablation (RFA), and the development of new ablation technologies such as mapping, cryoablation, and magnetic navigation, procedure-related acute complications have decreased (4–6), There remains a risk of complications after radiofrequency ablation (RFA).

Pericardial tamponade is one of the major complications of radiofrequency ablation (RFA). The incidence of pericardial tamponade in ventricular RFA is very low. Tokuda et al. statistically analyzed 1,152 RFA procedures for ventricular arrhythmias in a single center over 12 years and found that pericardial tamponade occurred in 11 cases, with an incidence of only 1% (7), but it carries high morbidity and mortality if unrecognized. Furthermore, tamponade can occur either intra-procedurally or as a delayed complication. Xu et al. reported a case of pericardial tamponade occurring 19 days after RFA for ventricular premature beats originating from the right ventricular outflow tract (8). Therefore, both operators and nursing staff need to be vigilant for the occurrence of pericardial tamponade during and after radiofrequency ablation (RFA).

The classical mechanism of tamponade is overt perforation, frequently related to steam-pop phenomena, in which tissue barotrauma from rapid intramyocardial steam formation disrupts the ventricular wall. The “explosive phenomenon” explains how energy delivery during RFA disrupts the heart wall: when the temperature at the interface between the ablation electrode and cardiac tissue rapidly rises above the boiling point, blood evaporates, causing small explosions and audible popping sounds. Evaporation may occur within the tissue, leading to the formation of intra-tissue air bubbles. As ablation proceeds, these bubbles expand and erupt through the weakest pathway, tearing the tissue. Intracardiac echocardiography can be used to identify the site where bubble explosions emit popping sound signals, which is often the site of cardiac perforation (9). O J Eick et al. conducted experiments using porcine ventricular tissue and found that the likelihood of popping significantly increases when the ablation electrode and endocardial tissue surface are exposed to flowing fluid and there is poor electrode-tissue contact. In these scenarios, tissue temperatures may be much higher than those measured at the tip electrode and may reach 100 °C, leading to intramyocardial steam formation and popping phenomena (10).

In our case, however, several features argue against an overt perforation: the absence of steam pop, limited volume of hemopericardium (150 ml), spontaneous hemostasis without surgical repair, negative biventricular angiography, and absence of effusion recurrence on serial echocardiography. Three possible mechanisms should be considered:

1. Classic perforation/steam pop—although not directly observed, this cannot be entirely excluded.
2. Small apical perforation with spontaneous sealing—previous study reliably detected a small confined region of the normal apical myocardium with a thickness of <3 mm—the left ventricular thin point (11); a minute defect may have temporarily bled and then sealed under pericardial pressure.
3. Thermal injury to epicardial or intramyocardial micro-vessels—heat-induced microvascular injury may represent a possible mechanism.

Alternative explanations, including structural abnormalities or coronary artery complications (spasm or dissection), also warrant consideration, although coronary angiography was not performed for safety reasons. This limitation, together with the absence of intracardiac echocardiography (ICE) or cardiac CT due to urgent and economic constraints, is acknowledged as a weakness of this report.

From a clinical perspective, several lessons emerge. First, early recognition is crucial: abrupt chest symptoms, fluoroscopic “halo” signs, and invasive pressure changes should prompt immediate suspicion of tamponade. Second, when drainage yields a small amount of blood with hemodynamic recovery and imaging excludes contrast leak, conservative management with indwelling catheter drainage can be sufficient. Third, careful modulation of ablation parameters in thin-walled ventricular regions may reduce the risk of microvascular rupture.

Traditional methods for identifying pericardial tamponade primarily involve clinical, radiological, and echocardiographic evaluations (12), fluoroscopy of the cardiac silhouette is very useful in radiofrequency ablation (RFA) (13), transthoracic echocardiography is the clinical diagnostic criterion for pericardial tamponade. Additionally, the application of new technologies helps accelerate diagnosis and treatment, van Roest MH et al. (14) used near-infrared spectroscopy (NIRS) to non-invasively monitor bilateral cerebral oxygen saturation. Before the patient's arterial blood pressure dropped, a sudden decrease in bilateral cerebral oxygen saturation was detected to identify pericardial tamponade, which was then diagnosed by transthoracic echocardiography, enabling early control of the complication. Finally, emerging pulsed field ablation (PFA) has shown a favorable safety profile with a lower risk of myocardial perforation and tamponade (15). As adoption increases, PFA may represent a safer alternative for selected patients.

## Conclusion

In summary, this case broadens the mechanistic spectrum of RFA-related tamponade beyond classic perforation. Awareness of potential causes, coupled with prompt recognition and tailored management, may improve outcomes in similar scenarios.

## Data Availability

The original contributions presented in the study are included in the article/Supplementary Material, further inquiries can be directed to the corresponding author.
