# Supplementary material for: Case Report: Pericardial tamponade during left ventricular radiofrequency ablation with spontaneous hemostasis
Source: Front Cardiovasc Med. 2025 Oct 29;12:1669648. doi: 10.3389/fcvm.2025.1669648 (PMC12605116; doi:10.3389/fcvm.2025.1669648)

Supplemental Figures

A: Preoperative cardiac ultrasound

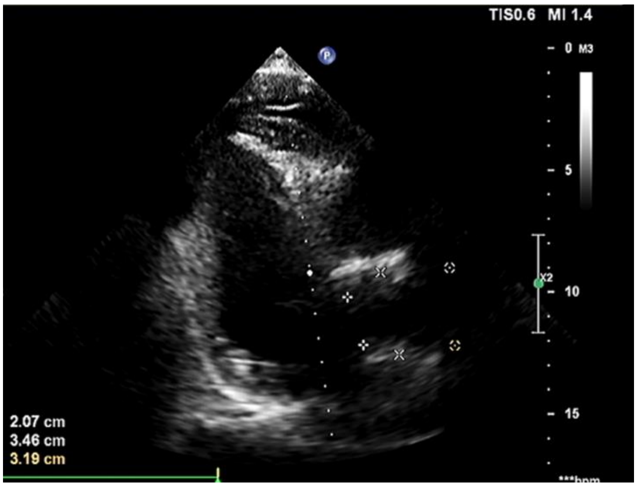

B Preoperative left ventricular myocardial contrast echocardiography

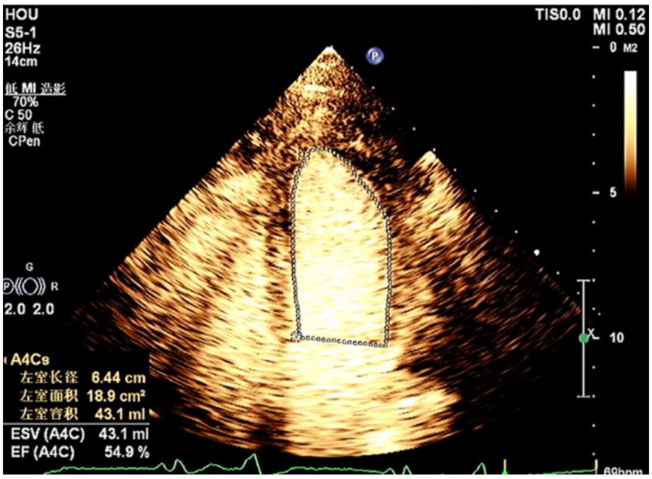

C: Intraoperative bedside cardiac ultrasound

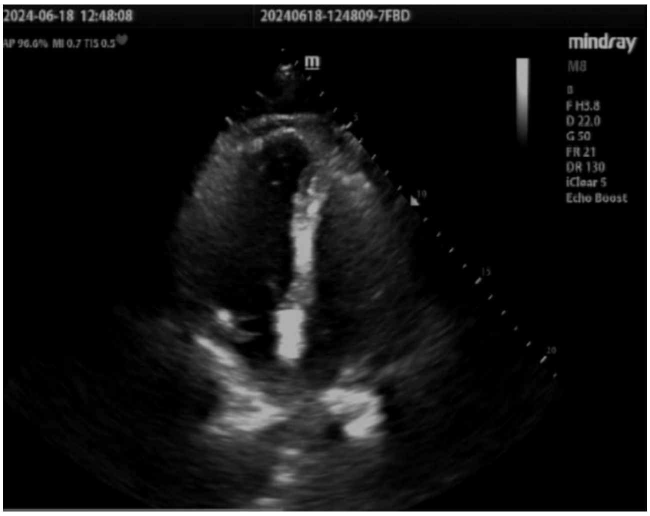

D Postoperative cardiac ultrasound

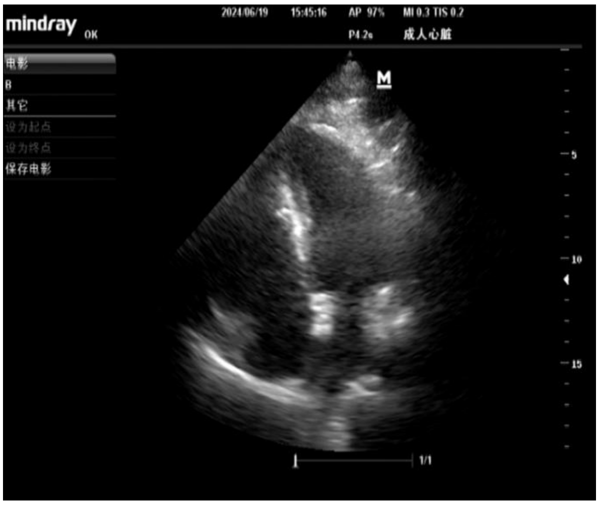

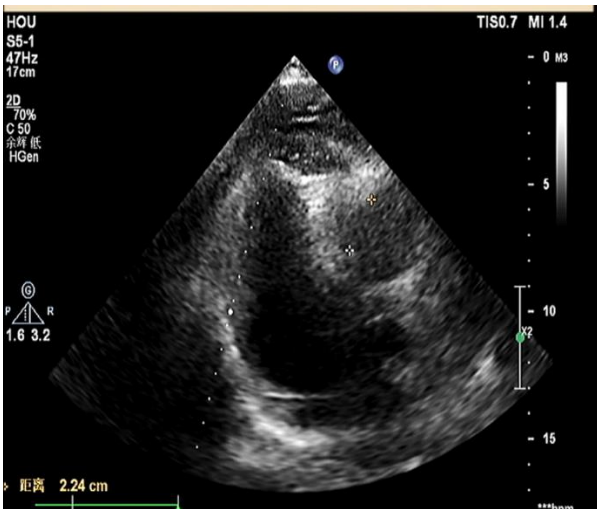

Supplement: Supplementary file 5 [file Datasheet1.pdf]
